# Supplementary material for: In-depth single molecule localization microscopy using adaptive optics and single objective light-sheet microscopy
Source: Nat Commun. 2025 Sep 24;16:8362. doi: 10.1038/s41467-025-62198-8 (PMC12460799; doi:10.1038/s41467-025-62198-8)
Supplement: Supplementary file 1 — Supplementary Information [file 41467_2025_62198_MOESM1_ESM.pdf]

# Supplementary Information

## In depth single molecule localization microscopy using adaptive optics and single objective light-sheet microscopy

Marine Cabillic[1,2#], Hisham Forriere[1#], Laetitia Bettarel[1], Corey Butler[1,3], Abdel Neuhaus[1], Ihssane Idrissi[1,2], Miguel Edouardo Sambrano-Lopez[1], Julian Rossbrioch[1], Lucas-Raphael Müller[4], Jonas Ries[4,5], Gianluca Grenci[6,7], Virgile Viasnoff[6], Florian Levet[1,8], Jean-Baptiste Sibarita[1]*, Rémi Galland[1]*

[1]*Univ. Bordeaux, CNRS, Interdisciplinary Institute for Neuroscience, IINS, UMR 5297, F-33000 Bordeaux, France*

[2]*Sanofi, Integrated Drug Discovery Department, Vitry-sur-Seine, Paris, France*

[3]*Imagine Optic, Orsay, France*

[4]*Cell Biology and Biophysics Unit, European Molecular Biology Laboratory (EMBL), Heidelberg, Germany*

[5] *Max Perutz Labs, Department of Structural and Computational Biology, University of Vienna, Vienna, Austria*

[6]*Mechanobiology Institute, National University of Singapore, Singapore*

[7]*Biomedical Engineering Department, National University of Singapore, Singapore*

[8]*Univ. Bordeaux, CNRS, INSERM, Bordeaux Imaging Center, BIC, UAR3420, US 4, F-33000 Bordeaux, France*

## Table of Contents

**SUPPLEMENTARY FIGURES** ........ **2**

SUPPLEMENTARY FIGURE 1 ........ 2
SUPPLEMENTARY FIGURE 2 ........ 5
SUPPLEMENTARY FIGURE 3 ........ 7
SUPPLEMENTARY FIGURE 4 ........ 8
SUPPLEMENTARY FIGURE 5 ........ 9
SUPPLEMENTARY FIGURE 6 ........ 10
SUPPLEMENTARY FIGURE 7 ........ 11
SUPPLEMENTARY FIGURE 8 ........ 12
SUPPLEMENTARY FIGURE 9 ........ 13
SUPPLEMENTARY FIGURE 10 ........ 14
SUPPLEMENTARY FIGURE 11 ........ 15
SUPPLEMENTARY FIGURE 12 ........ 16
SUPPLEMENTARY FIGURE 13 ........ 17
SUPPLEMENTARY FIGURE 14 ........ 18
SUPPLEMENTARY FIGURE 15 ........ 19

**SUPPLEMENTARY TABLES** ........ **20**

SUPPLEMENTARY TABLE 1: SMARt AND JeWell DEVICES DIMENSIONS ........ 20

**SUPPLEMENTARY MOVIE** ........ **21**

**SUPPLEMENTARY METHODS** ........ **22**

*AO module calibration and static aberrations correction* ........ *22*
*Light-Sheet characterization* ........ *22*
*Fixation and labelling in the SMARt devices for single cells imaging.* ........ *22*
*JeWell devices preparation and 3D cell cultures labelling* ........ *23*

**REFERENCES** ........ **23**

# Supplementary Figures

## Supplementary Figure 1

| Methods illustration & Reference | Excitation & Detection methods | Registration approach & Volumetric reconstruction | Pro & Cons |
|---|---|---|---|
| [1] 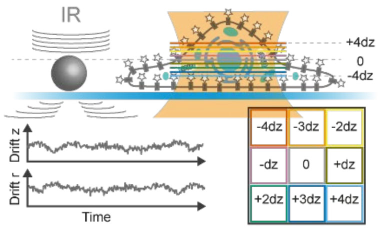  | • Widefield illumination for fluorescence excitation<br>• IR trans-illumination for latex beads detection<br><br>• Multi-focus Microscopy for 3D SM localization over axyally extended volume<br><br>• Obj: 100x, Oil, 1.4NA | • Real time active stabilization using IR detection of a non-fluorescent latex beads.<br><br>• Whole volume acquired at a time enabling accurate volumetric reconstruction.<br>• SM Loc.: 3D Gaussian fitting | ☺ Active drift stabilization<br>☺ Entire volume imaging at a time<br><br>☹ No optical sectioning<br>☹ No AO correction<br>☹ Restricted to single cell at the coverslips<br>☹ Restricted FOV |
| [2, 3] 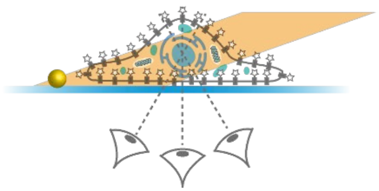 | • Hilo illumination.<br><br>• Light-field detection for 3D SM localization.<br><br>• Obj: 60x, WI, 1.27NA | • Light-field detection of a fiduciary located on the coverslip<br><br>• Whole volume acquired at a time enabling accurate volumetric reconstruction<br>• SM Loc.: Gaussian fiting and dedicated 3D assigment | ☺ Entire volume imaging at a time<br><br>☹ Limited optical sectioning<br>☹ No AO correction<br>☹ Restricted to single cell at the coverslips<br>☹ Restricted FOV |
| [4, 5] 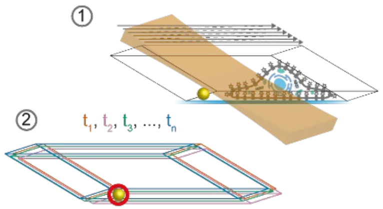  | • Light sheet excitation using a dithered lattice Light Sheet.<br><br>• WF astigmatism-based 3D SM localization using a cylindrical lens.<br><br>• Obj: 25x, WI, 1.1NA | • astigmatism-based 3D localization of fiduciary located on the coverslip.<br><br>[4]:<br>• Whole volume acquired continuously containing a fiduciary<br>- SM loc.: 2D Gausian fiting<br>[5]:<br>• Volumes composed of thousands of frames per plane acquired continusouly<br>- SM Loc.: CSpline fitting from interpolated experimental PSF | ☺ Very efficient optical sectioning<br><br>☹ No AO correction<br>☹ Complex set-up |

| Methods illustration & Reference | Excitation & Detection methods | Registration approach & Volumetric reconstruction | Pro & Cons |
|---|---|---|---|
| [6, 7]<br>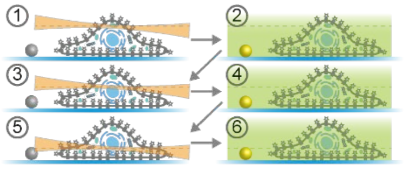 | • Tilted light sheet & widefield illuminaiton<br><br>• WF Double helix PSF detection using a phase mask or tetrapod PSF detection<br><br>• Obj: 100x, Oil, 1.4NA | • Interleaved SM and WF fiduciaries detection for active 3D registration<br><br>• Off-line drift compensation and slice stitching from fiduciaries detections & 3D Cross-correlation for final slice stitching.<br><br>• SM Loc.: Easy DHPSF software | ☺ Optical sectioning<br>☺ Possible active drift compensation<br><br>☹ No AO correction<br>☹ Low density SM regime<br>☹ Limited imaging depth |
| [8]<br>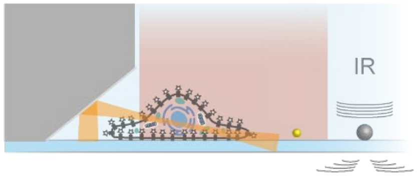 | • Light-sheet excitation after reflection of a dithered static-LS on a 39° mirror.<br>• IR trans-illumination or WF illumination for fiduciaries detection<br><br>• WF Double helix PSF detection<br><br>• Obj: 100x, Oil, 1.45NA | • Real time active stabilization using IR detection of a polystyrene bead.<br>• WF long axial range Double Helix PSF fiduciaries detection<br><br>• Off-line drift compensation and slice stitching from fiduciaries detections & 3D Cross-correlation for final slice stitching.<br><br>• SM Loc.: DECODE Deep-learning based localisation | ☺ Optical sectioning<br>☺ Active drift correction<br>☺ DL based high density regime SM localization<br><br>☹ No AO correction<br>☹ DHPSF sizes<br>☹ Limited imaging depth<br>☹ Dedicated imaging devices |
| This paper<br>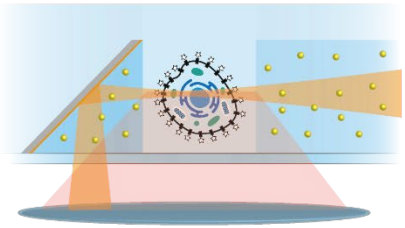 | • Light-sheet excitation after reflection of a scanning laser on a 45° mirror.<br><br>• WF astigmatism-based 3D SM localization using DM. Optical aberration correction using DM<br><br>• Obj: 60x, WI, 1.27NA | • WF astigmatism-based 3D localization of fiduciaries embedded in the device for real time drift compensation.<br><br>• Off-line drift compensation and slice stitching from fiduciaries detections<br><br>• SM Loc.: 2D Gaussian fitting & DECODE Deep-learning based localisation | ☺ Optical sectioning<br>☺ Active drift correction<br>☺ AO correction<br>☺ Imaging depth<br>☺ DL based high density regime SM localization<br><br>☹ Dedicated imaging devices<br>☹ No sample induced aberrations correction |

**Supplementary Figure 1: Comparison of volumetric 3D SMLM techniques.** Comparison of different published methods [1–8] for volumetric 3D SMLM.


1. Hajj, B. *et al.* Whole-cell, multicolor superresolution imaging using volumetric multifocus microscopy. *Proc. Natl. Acad. Sci. U. S. A.* **111**, 17480–17485 (2014).
2. Sims, R. *et al.* Single molecule light field microscopy. *Optica* **7**, 3 (2020).

3. Daly, S. *et al.* High-density volumetric super-resolution microscopy. *Nat. Commun.* **15**, 1940 (2024).
4. Legant, W. R. *et al.* High-density three-dimensional localization microscopy across large volumes. *Nat. Methods* **13**, 359–365 (2016).
5. Wäldchen, F. *et al.* Whole-cell imaging of plasma membrane receptors by 3D lattice light-sheet d STORM. *Nat. Commun.* **11**, 887 (2020).
6. Gustavsson, A.-K., Petrov, P. N., Lee, M. Y., Shechtman, Y. & Moerner, W. E. 3D single-molecule super-resolution microscopy with a tilted light sheet. *Nat. Commun.* **9**, (2018).
7. Nelson, T., Vargas-hernández, S., Freire, M., Cheng, S. & Gustavsson, A.-K. Multimodal illumination platform for 3D single-molecule super-resolution imaging throughout mammalian cells. *Biomed. Opt. Express* **15**, 3050 (2024).
8. Saliba, N., Gagliano, G. & Gustavsson, A. Whole-cell multi-target single-molecule super-resolution imaging in 3D with microfluidics and a single-objective tilted light sheet. *Nat Commun* **15**, 10187 (2024).
9. Lakshminarayanan, V. & Flece, A. TUTORIAL REVIEW - Zernike polynomials: A guide. *J. Mod. Opt.* **58**, 1678 (2011).
10. Zeng, J., Mahou, P., Schanne-Klein, M.-C., Beaurepaire, E. & Débarre, D. 3D resolved mapping of optical aberrations in thick tissues. *Biomed. Opt. Express* **3**, 1898 (2012).
11. Facomprez, A., Beaurepaire, E. & Débarre, D. Accuracy of correction in modal sensorless adaptive optics. *Opt. Express* **20**, 2598 (2012).
12. Beghin, A. *et al.* Automated high-speed 3D imaging of organoid cultures with multi-scale phenotypic quantification. *Nat. Methods* **19**, 881–892 (2022).
13. Grenci, G. *et al.* A High-Throughput Platform for Culture and 3D Imaging of Organoids. *J. Vis. Exp.* 1–22 (2022). doi:10.3791/64405

## Supplementary Figure 2

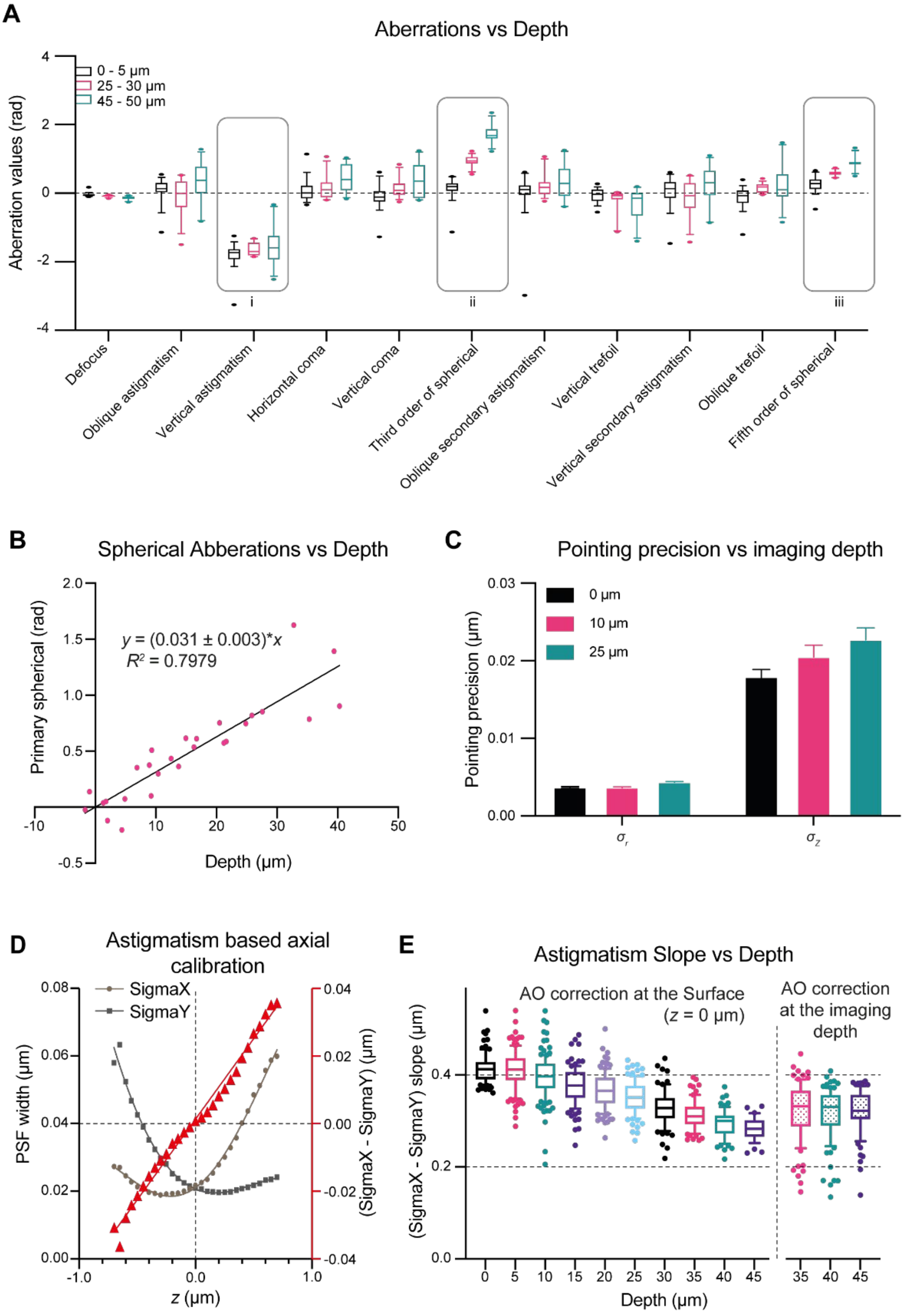


**Supplementary Figure 2: Characterization of depth-dependent aberrations.** (**A**) Aberrations retrieved from PSFs acquired using Tetraspeck fluorescent beads embedded in an agarose based-phantom sample at different depths (0-5 µm: $n = 18$; 25-30 µm: $n = 14$; 45-50 µm: $n = 11$ independent beads) (are represented: $mean \pm 10 - 90\ percentile$). (**B**) Characterization of the depth dependence of the 3rd order of the spherical aberration retrieved from PSFs acquired at different depths, using Tetraspeck fluorescent beads embedded in an agarose-based phantom sample ($n = 27$ beads). The black curve represents the linear regression curve. (**C**) Pointing precisions computed from images of Tetraspeck

fluorescent beads embedded in an agarose-based phantom sample. The pointing precision is computed from the distribution of 1,000 localizations per condition and retrieved from an astigmatism calibration performed at the coverslip (are represented: $mean \pm 95\% \ CI$). (**D**) Example of astigmatism calibration curve used for 3D localization, computed from images of Tetraspeck fluorescent beads embedded in an agarose-based phantom sample after aberration correction at the bead's depth. Width ($\sigma_x$) (brown circles) and height ($\sigma_y$) (dark grey squares) with their corresponding 2$^{nd}$ order polynomial fits, and $(\sigma_x - \sigma_y)$ (red triangles) with the corresponding linear regression curve used to compute the astigmatism slope used in **E**. (**E**) Slopes of the linear regression curve of $(\sigma_x - \sigma_y)$ computed from images of Tetraspeck fluorescent beads embedded in an agarose-based phantom sample acquired at different depth ($n = 84 \pm 19$ beads, $mean \pm s.e.m.$) (are represented: $mean \pm 10 - 90 \ percentile$). AO corrections were either applied at the coverslips surface (left) or at the imaging depth (right), followed by the application of 60 nm rms astigmatism for 3D localization.

## Supplementary Figure 3

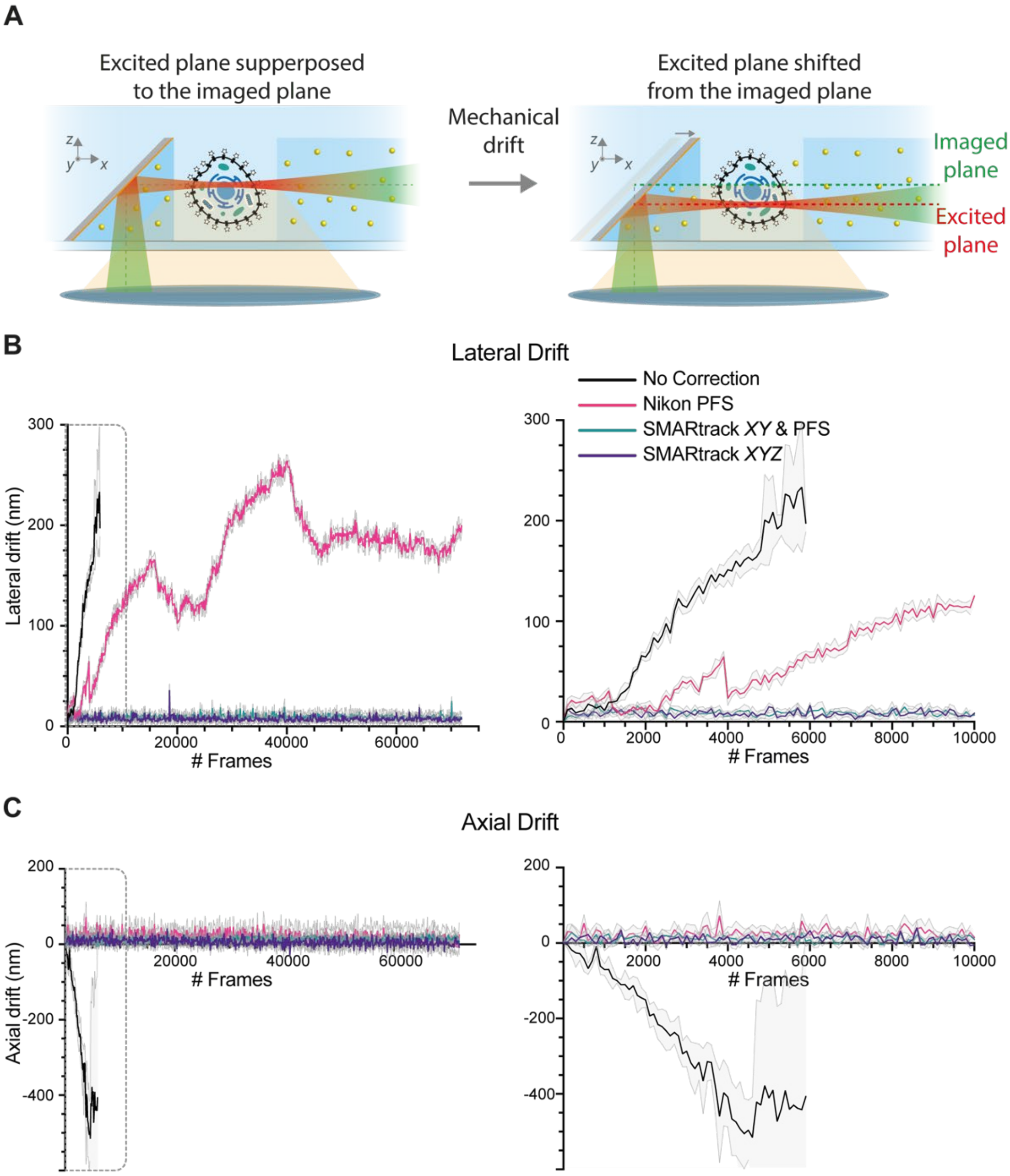


**Supplementary Figure 3: Real-time 3D drift correction.** (**A**) Schematic illustration of the relationship between the image and excitation planes due to the soSPIM architecture using 45° mirrors integrated into the imaging device to create the excitation light-sheet. Mechanical drifts along the x-axis induce an equivalent axial drift of the excitation plane according to the image plane. (**B**) Example of mechanical drifts measured in the lateral plane during an acquisition, in absence of correction (black), with a frame-by-frame axial drift correction using only the perfect focus system (PFS) of the Nikon microscope (magenta), and with the SMARtrack real-time drift correction process performed either only laterally (green), or both laterally and axially (purple). Right: Zoom on the first 10,000 frames of a total of 70,000 frames. The curves show the average relative localization and s.e.m. measured on 5 independent beads for each condition. (**C**) Same as (**B**) in the axial direction.

## Supplementary Figure 4

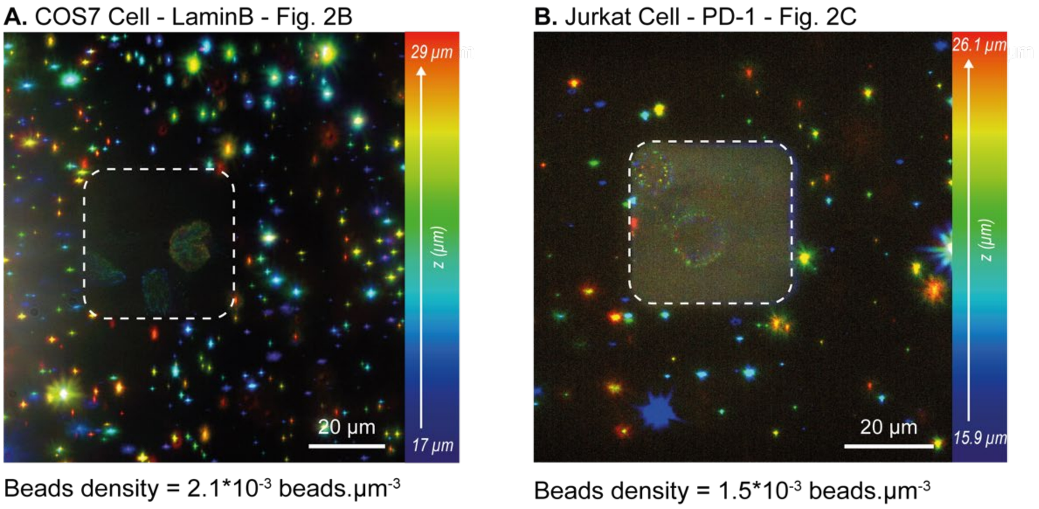


**Supplementary Figure 4: Fiduciary markers embedded in the soSMARt imaging devices.** (**A**) Maximum Intensity Projection (MIP) of a SMARt device field of view acquired in Fig. 2B, showing COS-7 cells labeled with Lamin B1, surrounded by fiduciary markers. The density of the fiduciaries embedded into the device polymer was estimated to be $2.1\ 10^{-3}\ \mu m^{-3}$. (**B**) Maximum Intensity Projection the SMARt device field of view acquired in Fig. 2C, showing Jurkat cells labeled with PD-1 receptors. The density of the fiduciaries embedded into the device polymer was estimated to be $1.5\ 10^{-3}\ \mu m^{-3}$. For all images, the well borders are indicated by dashed-lines, the colors represent the Z-distance to the coverslip, and brightness and gamma have been adjusted to visualize both the fiduciary markers and the single molecule signal within the well.

## Supplementary Figure 5

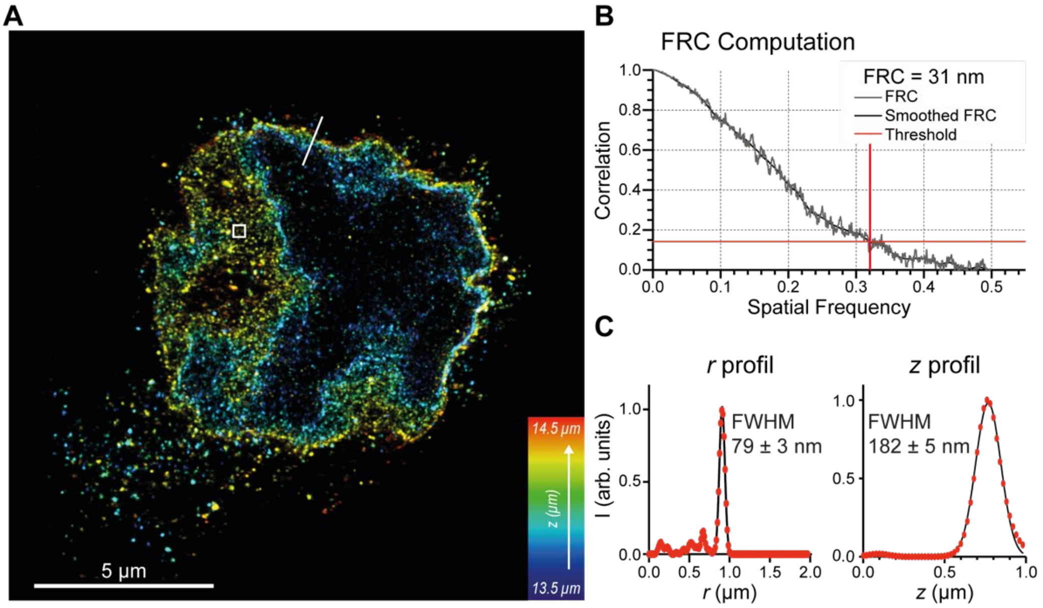


**Supplementary Figure 5: Spatial resolution at 14 µm depth.** (**A**) In-depth 3D SMLM reconstruction of a 1 µm thick optical section of the Lamin B1 nuclear envelope of a COS-7 cell acquired at 13.5 µm above the coverslip. Colors indicate the *z* distance from the coverslip. (**B**) Fourier ring correlation (FRC) curve computed from the image shown in (**A**), using two sub-image stacks reconstructed from even and odd localization identifiers with the BIOP/FRC ImageJ plugin (black and grey curves). The spatial resolution, defined by the 1/7 threshold criterion (red line), was estimated to $31\ \mathrm{nm}$. (**C**) Lateral and axial intensity profiles perpendicular to the Lamin envelope, with their corresponding FWHM obtained by Gaussian fitting. *Left*: XY-plane intensity profile along the white line in (A) with a FWHM of $79 \pm 3\ \mathrm{nm}$. *Right*: Z-axis intensity profile within the white square in (A), with a FWHM of $182 \pm 5\ \mathrm{nm}$.

## Supplementary Figure 6

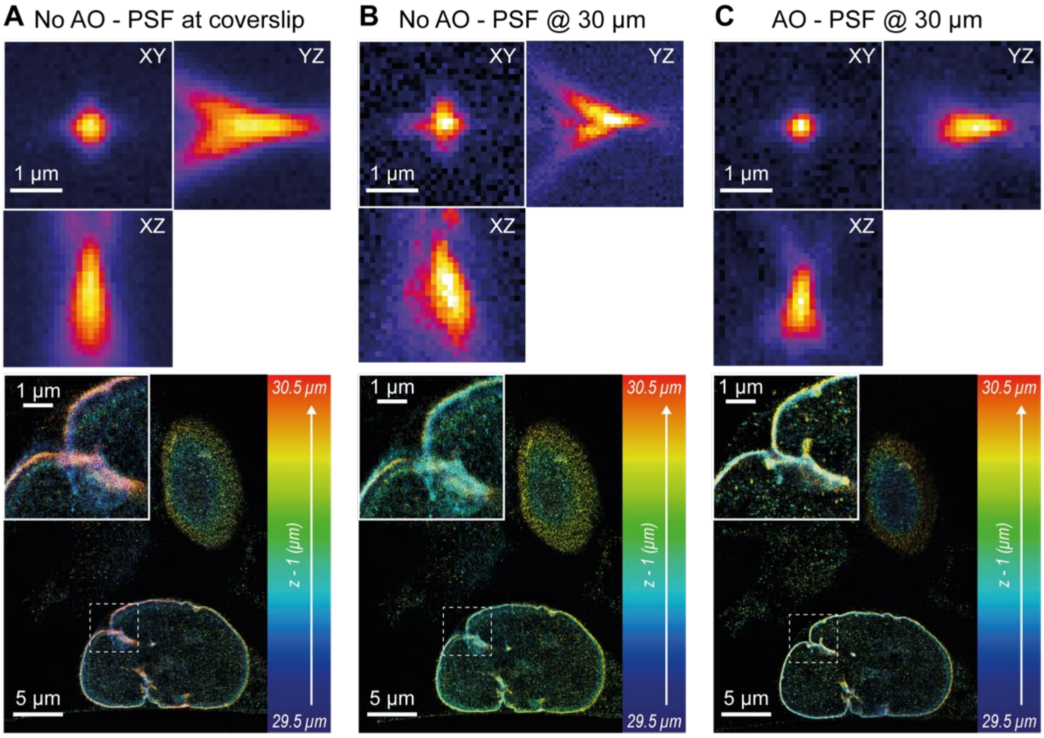


**Supplementary Figure 6: Comparison of reconstructions without and with AO.** (**A**) Top: Orthogonal views of an astigmatism-shaped PSF using a cylindrical lens, acquired at the coverslip. Bottom: 1 µm thick 3D reconstruction of the lamin B1 nuclear envelope acquired 30 µm above the coverslip, without aberration correction and using a cylindrical lens in the detection path. Reconstruction was performed using the astigmatism calibration curve performed on a PSF acquired at the coverslips (top). Inset: Zoom-in view of the region in the white dashed square. (**B**) Top: Orthogonal views of an astigmatism-shaped PSF using a cylindrical lens, acquired at the cell imaging depth, 30 µm above the coverslip. *Bottom*: 1 µm-thick 3D reconstruction of the lamin B1 nuclear envelope at the same depth, acquired without aberration correction and using a cylindrical lens. The reconstruction was performed using the astigmatism calibration performed using a PSF acquired at the imaging depth (top). Inset: Zoom-in view of the region in the white dashed square. (**C**) Top: Orthogonal views of an astigmatism-shaped PSF acquired 30 µm above the coverslip, following aberration correction and induction of 60 nm RMS astigmatism using the DM. Bottom: 1 µm thick 3D reconstruction of the lamin B1 nuclear envelope acquired at the same depth, following aberration correction and induction of 60 nm RMS astigmatism using the DM. The reconstruction was performed using the calibration curve obtained from the corrected PSF at the imaging depth (top). Inset: Zoom-in view of the region in the white dashed square. In all 3D reconstructions, colors indicate the *z* distance from the coverslip.

## Supplementary Figure 7

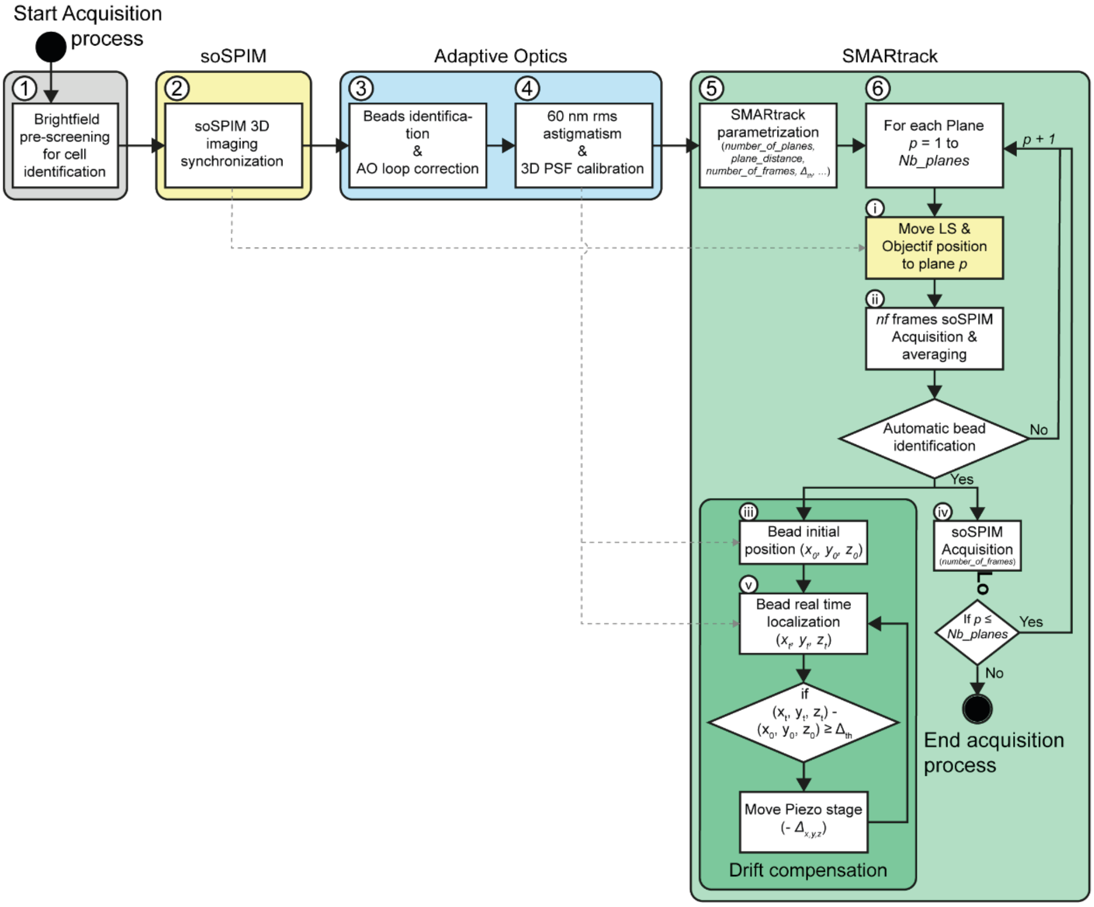


**Supplementary Figure 7: Acquisition process workflow.** The standard acquisition process starts with the acquisition of a large overview of the soSMARt device in brightfield mode, using the Scanslide module of MetaMorph acquisition software (1). It allows stitching multiple images to reconstruct a large field of view of the soSMARt device, and select the cell of interest. The soSPIM imaging system is then calibrated on the cell of interest field of view, by aligning and synchronizing the focal plane of the objective with the light-sheet position to ensure the superposition of the excitation and imaging planes at all depths (see Methods) (2). A single fiduciary marker positioned close to the volume of interest is selected and an AO 3N correction loop is performed using the bead Maximum Intensity as a metric (3). 60 nm rms of vertical astigmatism is then induced by the DM and a 3D PSF of the bead is acquired to calibrate the astigmatism-based 3D localization process (4). The SMARtrack software steers the whole acquisition process: First, the acquisition parameters are defined (*number_of_planes*, *plane_distance*, *number_of_frames* per plane, fiduciary markers selection criteria, drift correction distance threshold: $\Delta_{th}$, …) (5). Then, the acquisition process consists, for each plane $p$ ranging from 1 to *nb_of_planes*, to: (i) move the objective and the light-sheet to image plane $p$; (ii) acquire *nf* frames for fiduciary markers selection; (iii) Localize the fiduciary marker and memorize its initial 3D coordinates $(x_0, y_0, z_0)$ that will be used for feed-back loop registration; (iv) Perform the SMLM acquisition sequence of *nb_frames* and (v) the real time correction by tracking the displacements of the selected fiduciary marker. Drift correction is performed when $\left(x_f - x_0\right)^2 + \left(y_f - y_0\right)^2 + (z_f - z_0)^2 > \Delta_{th}^2$, by sending the drift to the *XYZ* piezo stage, with $(x_f, y_f, z_f)$ the 3D coordinates of the fiduciary at the frame $f$ of the SMLM acquisition sequence (6). The acquisition process is repeated until all the planes have been acquired. The dotted grey arrows represent the use of the initial calibrations (soSPIM, 3D PSF) during the volumetric acquisition process.

## Supplementary Figure 8

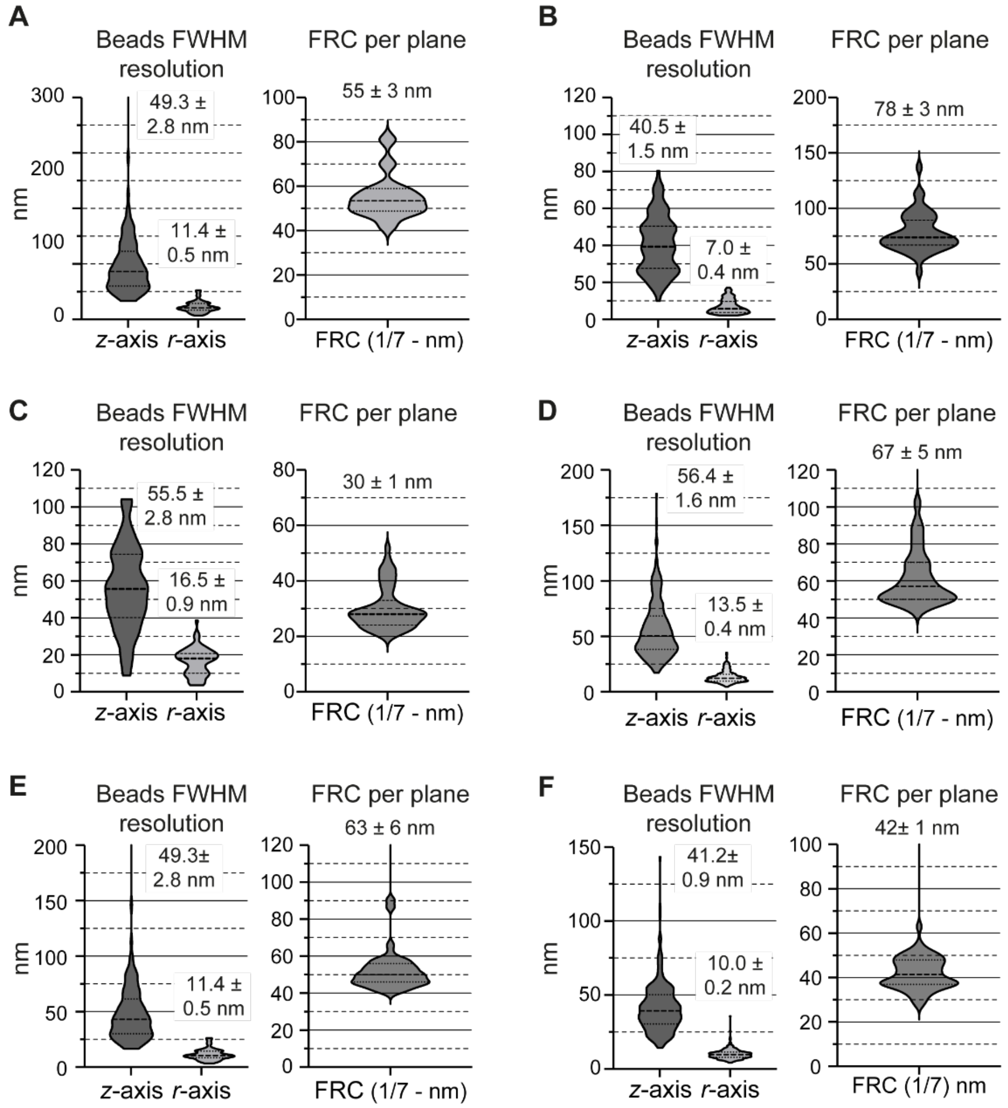


**Supplementary Figure 8: Spatial resolution estimation for volumetric 3D SMLM.**

(**A**-**F**) Spatial resolution estimation for the volumetric 3D SMLM acquisition represented in the different main and supplementary figures of the paper. For each panel: Left: Resolution estimated from the localization of fiduciary markers embedded in the SMARt device polymer, measured in the lateral (*r*-axis) and axial (*z*-axis) directions, from $n_b$ beads surrounding the well of interest (FWHM = 2.3 times the localization precision (Methods); $\mathrm{mean} \pm \mathrm{s.e.m.}$). Right: FRC-based spatial resolution ($\mathrm{mean} \pm \mathrm{s.e.m.}$) computed from the $n_p$ reconstructed plane, every 200 nm, of the entire acquired volume. (**A**) Acquisition of TOM20 in a COS-7 cell represented in Fig. 2A ($n_b = 110, n_p = 14$). (**B**) Acquisition of LaminB1 in a COS-7 cell represented in Fig. 2B ($n_b = 68, n_p = 55$). (**C**) Acquisition of PD-1 membrane receptors in a Jurkat T-cell represented in Fig. 2C ($n_b = 108, n_p = 45$). (**D**) Acquisition of CD3 membrane receptor on a Jurkat T-cell represented in Supp. Fig. 10A ($n_b = 217, n_p = 67$). (**E**) Acquisition of CD3 membrane receptor on a Jurkat T-cell represented in Supp. Fig. 10B ($n_b = 110, n_p = 81$). (**F**) Acquisition of LaminB1 in a COS-7 cell represented in Fig. 3A ($n_b = 299, n_p = 54$). Details of the computation of the resolution using beads' localization precision and FRC are provided in the Methods section.

## Supplementary Figure 9

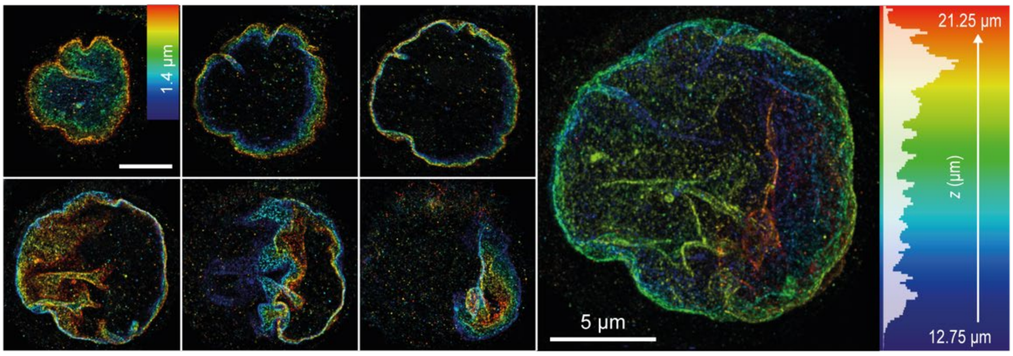


**Supplementary Figure 9: Volumetric 3D SMLM reconstruction of the entire Lamin B1 nuclear envelope of a COS-7 cell.** 3D reconstruction of the entire Lamin B1 nuclear envelope of a COS-7 cell suspended into a well of a *SMARt* device. Left: 1.4 µm thick reconstruction of the acquisition. Right: 3D reconstruction of the entire nucleus over 8.5 µm thickness, with the histogram of the number of localizations along the *z*-axis. Colors indicate the *z* distance from the coverslip. The histogram reveals distinct localization peaks corresponding to the optical planes, resulting from a too large interplane distance (500 nm in this case).

## Supplementary Figure 10

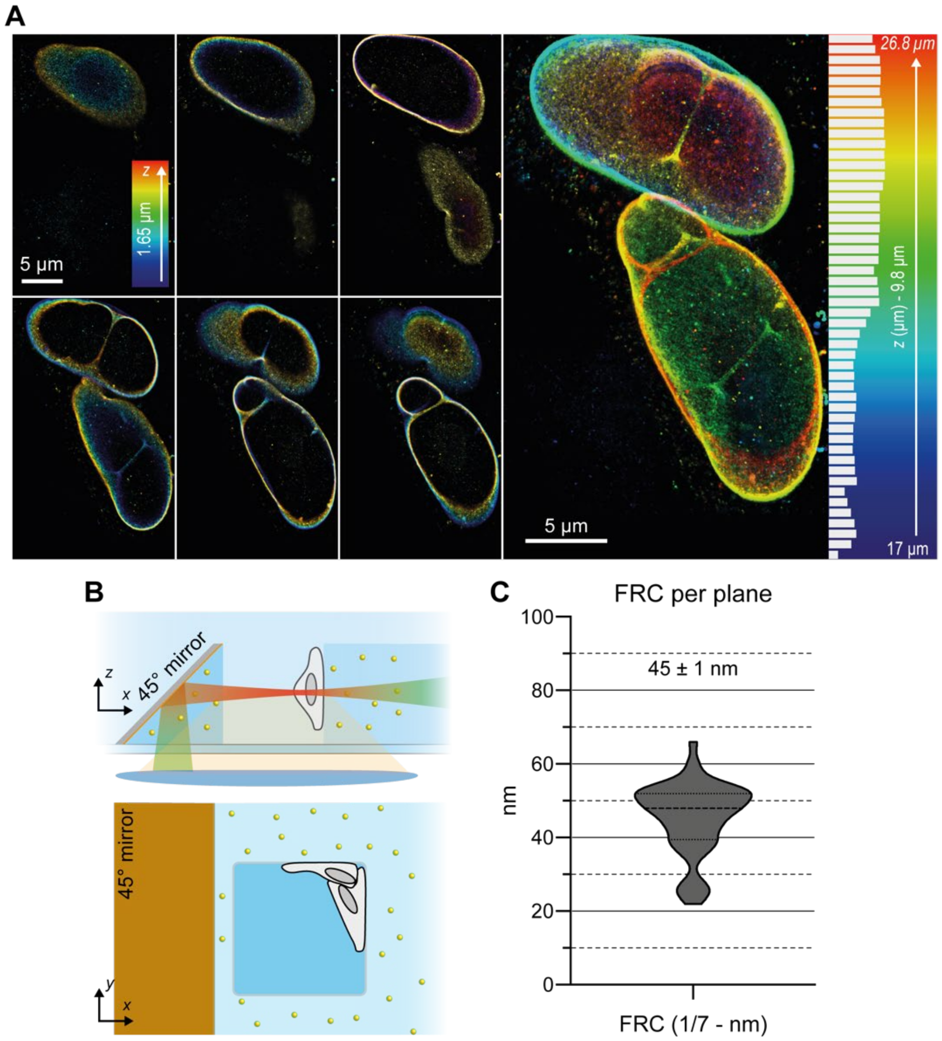


**Supplementary Figure 10: Volumetric 3D SMLM reconstruction of the Lamin B1 nuclear envelope of adherent COS-7 cells.** (**A**) 3D reconstruction of the Lamin B1 nuclear envelope of COS-7 cells cultured for 48h within a SMARt device. Left: 1.65 µm thick subvolume reconstruction. Right: Full 3D reconstruction of the nucleus over a depth of 9.8 µm, with the histogram of localization counts along the Z-axis. Colors indicate for the *z* distance from the coverslip. (**B**) Schematic illustration of the cell positioning, showing cells spread along the walls of a SMARt device well. (**C**) Spatial resolution estimation: $\mathrm{mean} \pm \mathrm{s.e.m.}$ of the FRC spatial resolution computed on each reconstructed plane across the entire volume ($n = 50$).

## Supplementary Figure 11

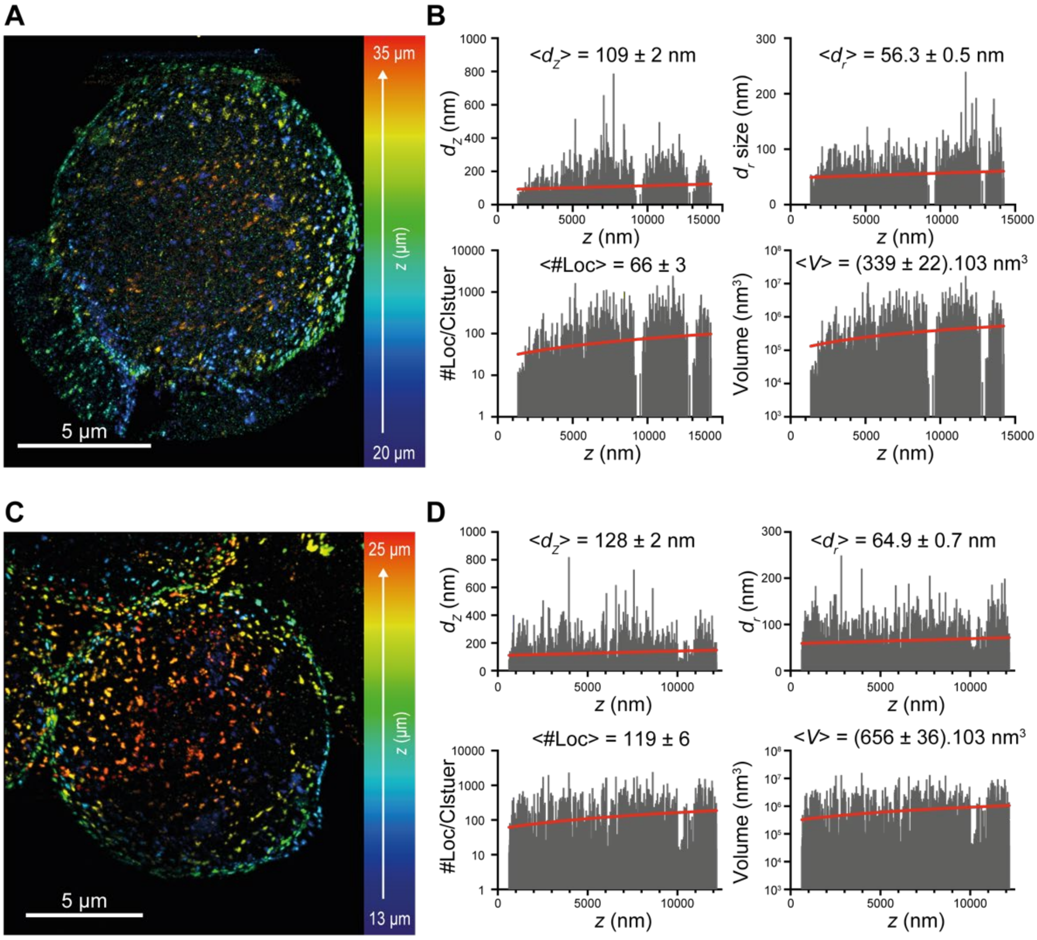


**Supplementary Figure 11: Quantification of the CD3 membrane receptors clusters on suspended Jurkat T-cell.** (**A & C**) 3D reconstruction of CD3 membrane receptors across the entire membrane of a Jurkat T-Cell suspended within a SMARt device well. Colors indicate the *z* distance from the coverslip. (**B**, resp. **D**) Quantification of the CD3 clusters properties computed from (**A**, resp. **C**) using the POCA clustering analysis software: FWHM along the axial ($d_z$) and lateral ($d_r$) directions, number of localizations (#Loc), volumes (*V*) (see Methods), as a function of the axial distance of the entire volume. Red curves show linear regressions for each parameter, highlighting the absence of depth-dependence bias in the quantifications ($R^2 \leq 0.024$). Values correspond to the $\text{mean} \pm \text{s.e.m.}$ for each cluster property ($n = 1853$ for **B**; $n = 1575$ for **D**).

## Supplementary Figure 12

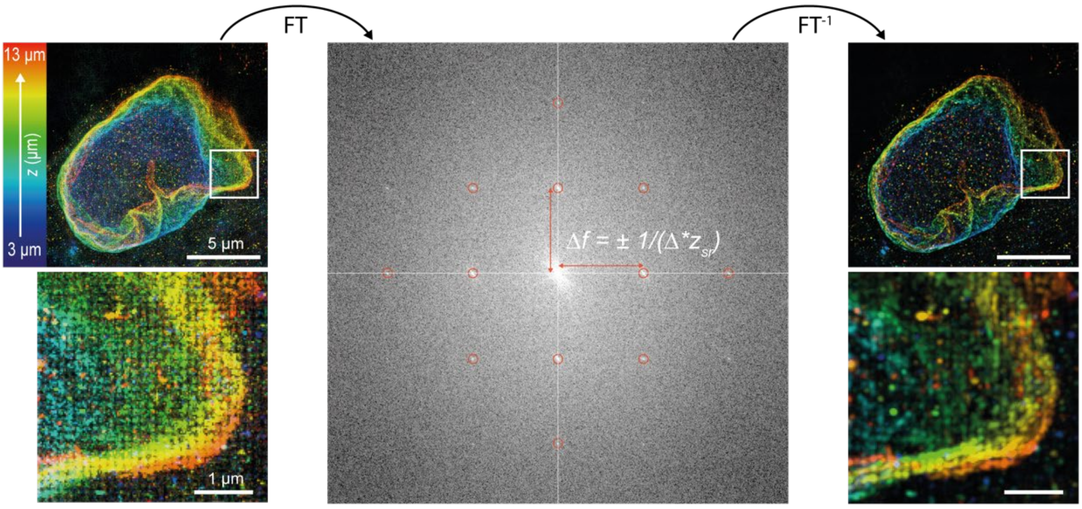


**Supplementary Figure 12: Grid pattern filtering for the deep learning-based reconstruction.** Left: 3D reconstruction of the Lamin B1 nuclear envelope of a COS-7 cell obtained using deep-learning-based DECODE single molecule localization. A zoom-in view of the white square region highlights the presence of a patterned artifact superimposed on the images. Middle: Fourier Transform (FT) of the deep-learning-based reconstruction. The red circles correspond to the spatial frequencies of the grid pattern observed in the reconstructions. These frequencies are located precisely at the multiples of $f = \pm 1/(\Delta * z_{sr})$ along both axes of the Fourier domain, where $\Delta$ the camera pixel size and $z_{sr}$ the zoom of the reconstructed image. Right: 3D reconstruction of the Lamin B1 nuclear envelope after Fourier filtering. Grid artifacts were removed by setting to zero a 3x3 pixel region centered a spatial the frequencies $f_{i=1..2} = \pm i/(\Delta * z_{sr})$ along both directions in the Fourier space. Colors indicate the *z* distance from the coverslip.

## Supplementary Figure 13

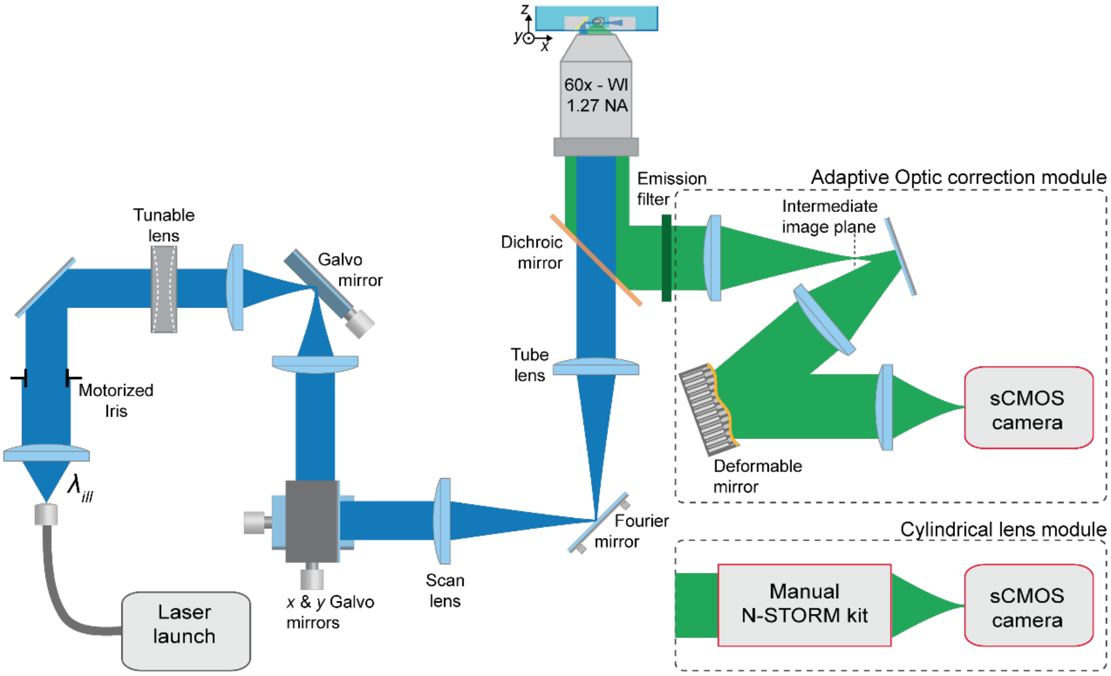


**Supplementary Figure 13: Schematic of the optical setup.** The laser beam ($\lambda_{ill}$) is sent to a tunable lens through a motorized iris to adjust the light-sheet (LS) parameters in the imaging plane (i.e., LS width and length). It is then directed to a pair of galvanometric mirrors, conjugated both to the tunable lens and to the objective back focal plane. The virtual conjugation of the two galvanometric mirrors to the objective back focal plane is performed thanks to a third galvanometric mirror positioned in the Fourier plane between the tunable lens and the galvanometric mirror pair. The beam if finally sent to the objective after reflection on a Fourier mirror conjugated to the objective image plane, which enables the adjustment of the LS pitch and tilt in the image plane. Fluorescence emission is collected either via a *4f* system incorporating a Deformable Mirror (DM) placed at the Fourier plane and imaged on a sCMOS camera, or via a commercial astigmatism module (N-STORM, Nikon) equipped with a cylindrical lens and imaged on a sCMOS camera. The schematic is not to scale.

## Supplementary Figure 14

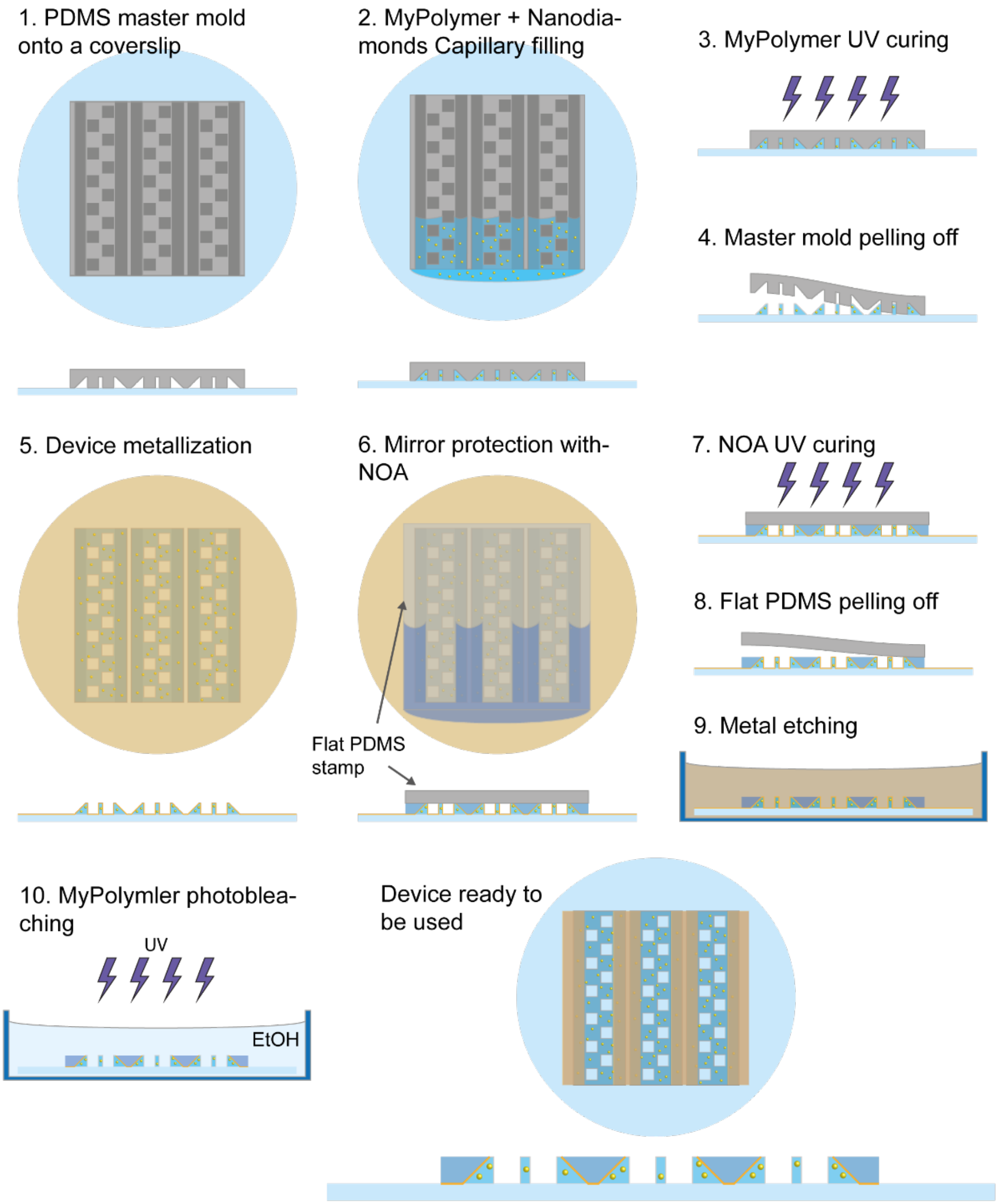


**Supplementary Figure 14: SMARt device fabrication process.** (**1**) A PDMS replica of a silicon master mold is positioned onto a coverslip and used to reproduced the master mold shapes (45° slanted surfaces and microwells) in a UV-curable polymer mixed with 90 nm fluorescence nanodiamonds via a capillary filling process (**2**, **3**). (**4**) Once the PDMS mold peeled off, the whole coverslip containing the structured polymer is metallized with a gold layer by thermal evaporation in vacuum (**5**). (**6** - **8**) The 45° mirrors is then protected by an additional layer of UV-curable polymer using a capillary filling process, thanks to the addition of a flat PDMS stamp onto the structured polymer. (**9**) The unprotected gold layer is then removed by wet etching. (**10**) Finally, the polymer autofluorescence is reduced by illuminating with UV the device immersed in Ethanol.

## Supplementary Figure 15

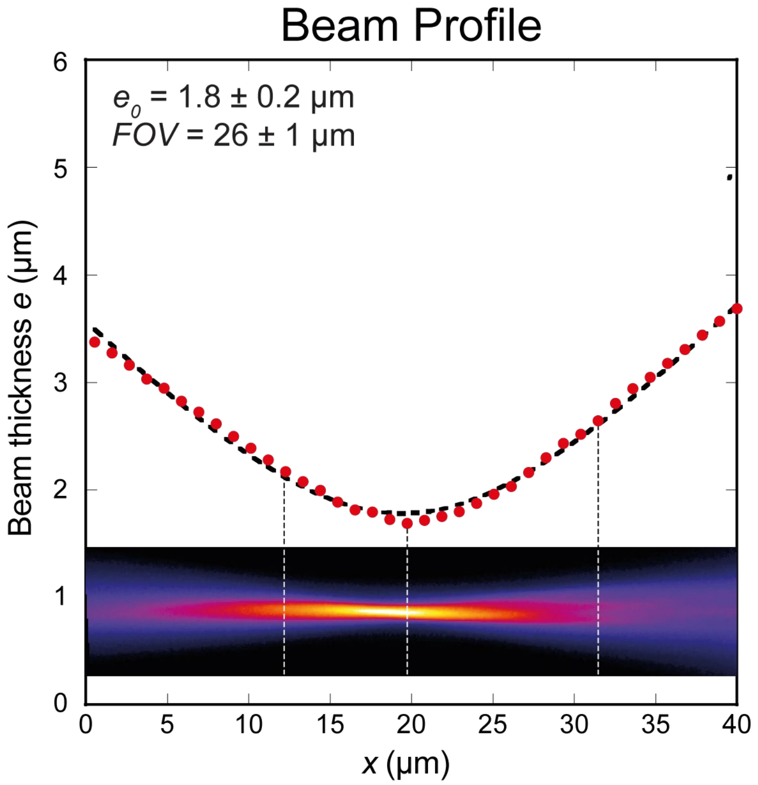


**Supplementary Figure 15: Beam profile characterization.** FWHM values extracted from intensity line profiles (white dotted lines) of the beam image acquired within a fluorescence solution (inset). These FWHM were plotted as a function of the beam position along the LS propagation (red dots). The beam width was fitted using the Gaussian beam propagation equation (black dotted line) (see Methods).

# Supplementary Tables

## Supplementary Table 1: SMARt and JeWell devices dimensions

| | Well size (µm x µm) | Well Height (µm) | Targeted samples |
|---|---|---|---|
| *SMARt* devices | 22 x 22 | 40 | Single cell and cells doublet |
| | 24 x 24 | | Single cell and cells doublet |
| | 40 x 40 | | Single cell, cells doublets and small cells aggregate |
| | 60 x 300 | | Small cells aggregate |
| JeWell devices | 270 x 270 | 100 | Spheroids, organoids |
| | 320 x 320 | | Spheroids, organoids |
| | 370 x 370 | | Spheroids, organoids |
| | 420 x 420 | | Spheroids, organoids |
| | 670 x 670 | 275 | Spheroids, organoids |

## Supplementary Movie

**Supplementary Movie 1:** Single molecule signal acquired at 14 µm depth after aberration correction and the addition of 60 nm rms astigmatism for 3D localization. It corresponds to the signal of the super-resolution reconstruction shown in Fig. 1E.

# Supplementary Methods

## AO module calibration and static aberrations correction

The AO module calibration and static aberrations correction of the system was performed using a sample containing both bright 1 µm fluorescent microbeads (Yellow-Green F8823, Thermofisher) and 0.1 µm fluorescent microbeads (Tetraspeck T7279, Thermofisher) adsorbed onto a 1.5H glass coverslip. First, the DM was calibrated using the SH-WFS mounted onto the AO module. A single 1 µm fluorescent microbeads is illuminated using the epifluorescence lamp in the Cy3 channel and used as a single point source as its size is smaller than the diffraction limit of the SH-WFS micro-lenses. Then, the WaveFronts (WF) are sequentially computed applying to each actuator of the DM a predefined positive and negative movements, enabling to build the DM/WF interaction matrix. A command matrix was computed based on singular value decomposition, establishing the relationship between the actuators' positions and the induced WF. It allowed to induce complex WF shapes such as the Zernike polynomials, which are widely used to represent optical aberrations [9]. As these Zernike polynomials are orthogonal, each mode can be induced with almost no crosstalk with each other, allowing sequential corrections. Each mode is expressed by its amplitude equivalent to the root mean square (rms) deviation from a flat WF. A first step correction of the system aberrations, called static aberrations, was performed in a close-loop mode using the SH-WFS. This correction step was considered as optimal when the remaining aberrations on the measured WF were below 20 nm rms, and used as a starting point for any further corrections.

In a second step, the static aberrations were further corrected using a "3N-algorithm" [10,11] by imaging a 0.1 µm fluorescent microbead, acting as a single point source, onto the sCMOS camera (Methods). It enabled us to determine the "flat" mirror shape, which corresponds to the mirror shape that cancels all the static aberrations of the system. It defines the initial DM shape prior depth-related aberrations corrections.

## Light-Sheet characterization

The Light-Sheet width (FWHM) and length (confocal parameter) used during the acquisition were measured to $1.8 \pm 0.2\ \mu\text{m}$ and $26 \pm 1\ \mu\text{m}$ $(\text{mean} \pm \text{s.e.m.})$, respectively (Supp. Fig. 14). These values were determined by imaging the beam after reflection onto the 45° mirror, without scanning, in a fluorescent solution (fluorescein, 1 µg/mL in water). Beam cross-section profiles were obtained by performing intensity line scans perpendicular to the direction of light propagation. Gaussian fittings of these profiles allowed retrieval of the beam thickness (FWHM) at multiple positions. The excitation beam profile was then reconstructed by plotting the beam thickness versus light propagation distance, and fitted using a Gaussian beam-propagation equation below, to retrieved the beam width and length.

$$\omega(x) = \omega_0 \sqrt{1 + \left(\frac{x - x_0}{Z_R}\right)^2} \quad (1)$$

Here, $\omega_0$ denotes the beam waist, representing the minimum thickness of the light sheet; $Z_R$ is he Rayleigh length, corresponding to half the FOV of the light sheet; and $x_0$ indicates the position of the beam focus along the light-sheet propagation.

## Fixation and labelling in the SMARt devices for single cells imaging.

Fixation and labelling steps were directly performed within the petri dishes containing the SMARt coverslip. For fixation, the devices were washed two times with pre-warmed PBS and incubated for 10 min in 4% (resp. 2%) paraformaldehyde-sucrose solution (ThermoFisher, #28906) for COS7 cells (resp. Jurkat cells). After 3 rinsing steps with PBS, the cells were quenched in 150 mM of Glycine (#26-128-6405-C, Euromedex) for 10 min and, in the case of intra cellular organelle labelling (Lamin, TOM20),

permeabilized for 10 min in 0.3 % triton X-100 (#T-9584, Sigma). Cells were then blocked for 2h at RT in 3 % BSA (#A3059, Sigma) in PBS supplemented with 0.1 mg/mL of sheared salmon sperm (#AM9680, Invitrogen) and 0.1% w/v of dextran sulfate (#D4911, Sigma). For the immunostaining steps, 200 µL of primary antibodies diluted in the blocking solution (1:200) was then deposited onto the wells and allowed to incubate for 2h at RT (resp. overnight at 4°C) for COS7 cells (resp. Jurkat cells) (anti-LaminB1 (Rb): ab16048 abcam; anti-TOM20 (Ms, clone 4F3): ab56783 abcam; PD-1 anti-Human (Mouse, clone MIH4), #14-99969-82, Invitrogen; CD3 anti-Human (Mouse, clone OKT3), #16-0037-81, eBioscience) followed by 3 washing steps with the blocking solution with 5min intervals of resting time between each washing step. 200 µL of the appropriate secondary antibodies (anti-Ms-$D_1$ or anti-Rb-$D_2$) labelled with DNA docking strands for DNA-PAINT imaging (Massive-sdAB-2 Plex, MASSIVE Photonics) diluted in the blocking solution (1:200) was finally deposited onto the wells and allowed to incubate for 1h (resp. 3h) at room temperature for COS7 cells (resp. Jurkat cells). The device was finally washed three times with the blocking solution followed by three times with PBS and kept in PBS at 4°C prior imaging. For Jurkat cells, a post-fixation step was performed for 5 min in 2% PFA-sucrose solution.

### JeWell devices preparation and 3D cell cultures labelling

**JeWells fabrication and preparation.** The JeWells microfabrication process was achieved as described in [12] and [13]. Once fabricated, JeWells were passivated using lipidure coating (Lipidure-CM5206, NOF Europe) to avoid cell adhesion into the JeWells surface, and therefore allow spheroids formation and culture. Briefly, 2 mL of Ethanol was poured onto the Jewell prior to be outgazed to remove any bubble trapped into the pyramidal wells. The Ethanol was then removed and replaced by 1 mL of a lipidure solution diluted in pure ethanol (0.5% w/v). The lipidure solution was then aspirated and the device left to dry under a sterile hood in order to form a thin anti-adhesive layer all over the device's surfaces. Just before cell seeding, the device was immersed in PBS, outgazed, sterilized under UV for 15 min and finally filled with culture media and kept in a sterile environment.

**Fixation and labelling.** Fixation and labelling steps were directly performed within the JeWell devices glued onto bottom-free petri-dishes. For fixation, the JeWell devices were rinsed two times with sterile PBS, and the 3D cell cultures were fixed for 20 min in 4% paraformaldehyde at room temperature. The 3D cultures were then quenched in a 150 mM Glycine (Sigma) solution for 2h and permeabilized for 2h in 0.3% Triton-X-100 (T#9284, Sigma-Aldrich) solution in sterile PBS at room temperature, followed by an overnight incubation in blocking solution (3% bovine serum albumin BSA in sterile PBS). Samples were then incubated with 1:1000 anti-laminB1(Rb) antibody (ab16048) diluted in blocking solution supplemented with 0.1% Triton-X-100 at 4 °C overnight followed by three rinsing steps with blocking buffer. Finally, Samples were incubated with 0.5 µg/ml DAPI (#62248, ThermoFisher) and 1:500 anti-Rab-$D_2$ secondary antibodies diluted in blocking solution supplemented with 0.1% Triton-X-100 for 4h at room temperature, followed by three rinsing steps with blocking buffer and three rinsing steps with sterile PBS before to be stored at 4°C prior imaging.

# References


1. Hajj, B. *et al.* Whole-cell, multicolor superresolution imaging using volumetric multifocus microscopy. *Proc. Natl. Acad. Sci. U. S. A.* **111**, 17480–17485 (2014).
2. Sims, R. *et al.* Single molecule light field microscopy. *Optica* **7**, 3 (2020).
3. Daly, S. *et al.* High-density volumetric super-resolution microscopy. *Nat. Commun.* **15**, 1940 (2024).
4. Legant, W. R. *et al.* High-density three-dimensional localization microscopy across large volumes. *Nat. Methods* **13**, 359–365 (2016).
5. Wäldchen, F. *et al.* Whole-cell imaging of plasma membrane receptors by 3D lattice light-sheet d STORM. *Nat. Commun.* **11**, 887 (2020).

6. Gustavsson, A.-K., Petrov, P. N., Lee, M. Y., Shechtman, Y. & Moerner, W. E. 3D single-molecule super-resolution microscopy with a tilted light sheet. *Nat. Commun.* **9**, (2018).

7. Nelson, T., Vargas-hernández, S., Freire, M., Cheng, S. & Gustavsson, A.-K. Multimodal illumination platform for 3D single-molecule super-resolution imaging throughout mammalian cells. *Biomed. Opt. Express* **15**, 3050 (2024).

8. Saliba, N., Gagliano, G. & Gustavsson, A. Whole-cell multi-target single-molecule super-resolution imaging in 3D with microfluidics and a single-objective tilted light sheet. *Nat Commun* **15**, 10187 (2024).

9. Lakshminarayanan, V. & Flece, A. TUTORIAL REVIEW - Zernike polynomials: A guide. *J. Mod. Opt.* **58**, 1678 (2011).

10. Zeng, J., Mahou, P., Schanne-Klein, M.-C., Beaurepaire, E. & Débarre, D. 3D resolved mapping of optical aberrations in thick tissues. *Biomed. Opt. Express* **3**, 1898 (2012).

11. Facomprez, A., Beaurepaire, E. & Débarre, D. Accuracy of correction in modal sensorless adaptive optics. *Opt. Express* **20**, 2598 (2012).

12. Beghin, A. *et al.* Automated high-speed 3D imaging of organoid cultures with multi-scale phenotypic quantification. *Nat. Methods* **19**, 881–892 (2022).

13. Grenci, G. *et al.* A High-Throughput Platform for Culture and 3D Imaging of Organoids. *J. Vis. Exp.* 1–22 (2022). doi:10.3791/64405
