# Supplementary material for: In-depth single molecule localization microscopy using adaptive optics and single objective light-sheet microscopy
Source: Nat Commun. 2025 Sep 24;16:8362. doi: 10.1038/s41467-025-62198-8 (PMC12460799; doi:10.1038/s41467-025-62198-8)
Supplement: Supplementary file 2 — Description of Additional Supplementary Files [file 41467_2025_62198_MOESM2_ESM.pdf]

Description of Additional Supplementary Files

Supplementary Movie 1: Single molecule signal acquired at 14 µm depth after aberration correction and the addition of 60 nm rms astigmatism for 3D localization. It corresponds to the signal of the super-resolution reconstruction shown in Fig. 1E.
