# Supplementary material for: In-depth single molecule localization microscopy using adaptive optics and single objective light-sheet microscopy
Source: Nat Commun. 2025 Sep 24;16:8362. doi: 10.1038/s41467-025-62198-8 (PMC12460799; doi:10.1038/s41467-025-62198-8)
Supplement: Supplementary file 4 — Reporting Summary [file 41467_2025_62198_MOESM4_ESM.pdf]

nature portfolio

Corresponding author(s): Remi Galland

Last updated by author(s): 19/06/2025

Reporting Summary

Nature Portfolio wishes to improve the reproducibility of the work that we publish. This form provides structure for consistency and transparency in reporting. For further information on Nature Portfolio policies, see our Editorial Policies and the Editorial Policy Checklist.

Statistics

For all statistical analyses, confirm that the following items are present in the figure legend, table legend, main text, or Methods section.

n/a	Confirmed	
[ ]	[x]	The exact sample size (*n*) for each experimental group/condition, given as a discrete number and unit of measurement
[x]	[ ]	A statement on whether measurements were taken from distinct samples or whether the same sample was measured repeatedly
[x]	[ ]	The statistical test(s) used AND whether they are one- or two-sided<br>*Only common tests should be described solely by name; describe more complex techniques in the Methods section.*
[x]	[ ]	A description of all covariates tested
[x]	[ ]	A description of any assumptions or corrections, such as tests of normality and adjustment for multiple comparisons
[ ]	[x]	A full description of the statistical parameters including central tendency (e.g. means) or other basic estimates (e.g. regression coefficient) AND variation (e.g. standard deviation) or associated estimates of uncertainty (e.g. confidence intervals)
[x]	[ ]	For null hypothesis testing, the test statistic (e.g. *F*, *t*, *r*) with confidence intervals, effect sizes, degrees of freedom and *P* value noted<br>*Give P values as exact values whenever suitable.*
[x]	[ ]	For Bayesian analysis, information on the choice of priors and Markov chain Monte Carlo settings
[x]	[ ]	For hierarchical and complex designs, identification of the appropriate level for tests and full reporting of outcomes
[x]	[ ]	Estimates of effect sizes (e.g. Cohen's *d*, Pearson's *r*), indicating how they were calculated

Our web collection on statistics for biologists contains articles on many of the points above.

Software and code

Policy information about availability of computer code

Data collection	Data were acquired using the software MetaMorph from Molecular Device (version: 7.10.3.288).<br>The soSPIM beam steering unit were steered using the home made soSPIM plugin of MetaMorph availbale at: https://github.com/jbsiba/soSPIM<br>The SMARtrack solution used to actively correct for the mechanical drifts and for the acquisition automation is a plugin of MetaMorph available at: https://github.com/jbsiba/ZTrack<br>Optical aberrations correction and control of the Deformable Mirror were performed using the MicAO 1.3 software from Imagine Optics
Data analysis	Data were analyzed with the PALMTracer plugin (2019.12) of the MetaMorph software (version: 7.10.3.288) that is available upon request.<br>Data were represented using the ThunderSTORM plugin (1.3-2014-11-08) of ImageJ (1.53t), and FRC computation were performed using the Image Analysis tools of the BIOP plugin of ImageJ.<br>Data Clustering and clusters analysis works were performed using the POCA software available at https://github.com/flevet/PoCA<br>Graph and statistical analysis were performed using the software GraphPad PRISM 10.4.1 (532)<br>Artefactual periodical structure removal on high density SMLM data set reconstruction were performed using the ImageJ macro available at: https://github.com/jbsiba/SR_FFT_Filtering

For manuscripts utilizing custom algorithms or software that are central to the research but not yet described in published literature, software must be made available to editors and reviewers. We strongly encourage code deposition in a community repository (e.g. GitHub). See the Nature Portfolio guidelines for submitting code & software for further information.

Data

Policy information about availability of data

All manuscripts must include a data availability statement. This statement should provide the following information, where applicable:

- Accession codes, unique identifiers, or web links for publicly available datasets
- A description of any restrictions on data availability
- For clinical datasets or third party data, please ensure that the statement adheres to our policy

The single-molecule localization data generated and analyzed in this study, as well as the source data used to produce the graphs, have been deposited in the Zenodo database under accession code [https://zenodo.org/records/15168650].
Reference: Cabillic M, Forriere H, Bettarel L, et al. soSMARt: In depth single molecule localization microscopy using adaptive optics and smart single objective light-sheet microscopy. April 2025. doi:10.5281/zenodo.15168650

Research involving human participants, their data, or biological material

Policy information about studies with human participants or human data. See also policy information about sex, gender (identity/presentation), and sexual orientation and race, ethnicity and racism.

Reporting on sex and gender	N.A.
Reporting on race, ethnicity, or other socially relevant groupings	N.A.
Population characteristics	N.A.
Recruitment	N.A.
Ethics oversight	N.A.

Note that full information on the approval of the study protocol must also be provided in the manuscript.

Field-specific reporting

Please select the one below that is the best fit for your research. If you are not sure, read the appropriate sections before making your selection.

☒ Life sciences ☐ Behavioural & social sciences ☐ Ecological, evolutionary & environmental sciences

For a reference copy of the document with all sections, see nature.com/documents/nr-reporting-summary-flat.pdf

Life sciences study design

All studies must disclose on these points even when the disclosure is negative.

Sample size	No statistical sample size calculation was performed, as this study focuses on the development and validation of an imaging method rather than on testing biological hypotheses. Consequently, sample sizes were determined empirically based on technical considerations and reproducibility of the imaging performance.<br>In cases where variability was observed, it reflected physical or optical phenomena (e.g., depth-dependent aberrations), not biological variation. Therefore, a limited number of measurements was sufficient to characterize and illustrate these effects reliably.
Data exclusions	Single Molecules localizations for all presented datasets were filtered based on quality metrics (i.e. goodness of fit 〖: χ〗 ^2∈[0.6;1] and axial localization range: z∈[-0.5;0.5]) in order to only keep the best localizations and avoid as much as possible any reconstruction artefacts as described in the Methods sections.
Replication	This study presents a new imaging method and does not report any new biological discoveries.<br>Reproducibility of the methodology was assessed through multiple independent SMLM acquisitions of entire cells, as shown in both the main and supplementary figures of the manuscript.
Randomization	This study does not involve allocation into experimental groups, as it does not include any biological comparison or hypothesis testing. The work focuses on the development and technical validation of an imaging method. Sample grouping or randomization was therefore not applicable.
Blinding	No blinded acquisition or analysis was performed, as this study focuses on the development and validation of a new imaging method rather than on testing a biological hypothesis. Blinding was therefore not relevant in this context, given that no new biological findings were reported or evaluated.

Reporting for specific materials, systems and methods

We require information from authors about some types of materials, experimental systems and methods used in many studies. Here, indicate whether each material, system or method listed is relevant to your study. If you are not sure if a list item applies to your research, read the appropriate section before selecting a response.

Materials & experimental systems

n/a	Involved in the study
☐	☒ Antibodies
☐	☒ Eukaryotic cell lines
☒	☐ Palaeontology and archaeology
☒	☐ Animals and other organisms
☒	☐ Clinical data
☒	☐ Dual use research of concern
☒	☐ Plants

Methods

n/a	Involved in the study
☒	☐ ChIP-seq
☒	☐ Flow cytometry
☒	☐ MRI-based neuroimaging

Antibodies

Antibodies used

Primary antibodies used in this study included:
Anti-Lamin B1 (rabbit polyclonal, Abcam, ab16048; no clone - Lot:GR3383070-1) - dilution used 1:200 (single cells) to 1:1000 (3D cell cultures)
Anti-TOMM20 (mouse monoclonal, Abcam, ab56783; clone 4F3 - Lot:GR3382368-1) - dilution used 1:200
Anti-human PD-1 (mouse monoclonal, Invitrogen, #14-99969-82; clone MIH4) - dilution used 1:200
Anti-human CD3 (mouse monoclonal, eBioscience, #16-0037-81; clone OKT3) - dilution used 1:200

Secondary antibodies used:
Anti-Mouse-D1, Massive-sdAB-2 Plex (MASSIVE Photonics; single-domain antibody, no clone) - dilution used 1:200
Anti-Rabbit-D2, Massive-sdAB-2 Plex (MASSIVE Photonics; single-domain antibody, no clone) - dilution used 1:200
All antibodies were used according to the manufacturers' recommendations. Clones are indicated where applicable; polyclonal or sdAB-based antibodies are noted accordingly.

Validation

All primary antibodies used in this study were commercially available and validated by the respective manufacturers for immunofluorescence applications. Below is a summary of validation sources:
- Anti-Lamin B1 (rabbit polyclonal, Abcam, ab16048): Validated by Abcam for IF and WB applications in multiple species.
- Anti-TOMM20 (mouse monoclonal, Abcam, ab56783; clone 4F3): Validated for IF and WB by Abcam across multiple cell types. Widely cited in the literature.
- Anti-human PD-1 (mouse monoclonal, Invitrogen, #14-99969-82; clone MIH4): Validated by the manufacturer for flow cytometry and immunofluorescence on human samples. The clone MIH4 is well-established in the literature for detecting PD-1 expression on T cells.
- Anti-human CD3 (mouse monoclonal, eBioscience, #16-0037-81; clone OKT3): Validated by the manufacturer for flow cytometry and IF applications.The clone OKT3 is a widely used and well-characterized antibody in the literature.

No additional in-house antibody validation was performed beyond comparison with known subcellular localization patterns, which were consistent with expected staining.

Eukaryotic cell lines

Policy information about cell lines and Sex and Gender in Research

Cell line source(s)

Cell lines used in this study:
- COS-7 cells (African green monkey kidney fibroblast-like cell line) were obtained from ECACC, via Sigma-Aldrich (Catalog #87021302) - Sex: female (original source: female monkey).
- Jurkat cells (human T lymphocyte cell line) were purchased from ATCC, clone E6-1 (Catalog #TIB-152) - Sex: male (derived from a male donor).
- HepG2 H2B-GFP cells (human hepatocellular carcinoma cell line stably expressing H2B-GFP) were provided by the laboratory of F. Saltel. These cells were generated by lentiviral transduction using an LV H2B-GFP vector also produced by the Saltel lab - Sex: male (HepG2 is derived from a male donor).

Authentication

- COS-7 and Jurkat (clone E6-1): No in-house authentication was performed for these cell lines. Both were obtained directly from certified cell banks.
- HepG2::H2B-GFP: The HepG2 cell line (ATCC HB-8065) is a well-established human hepatocellular carcinoma line authenticated by ATCC using STR profiling. The HepG2::H2B-GFP line was generated in the laboratory of F. Saltel via lentiviral transduction. No additional authentication was performed.

Mycoplasma contamination

All cell lines were regularly tested negative for mycoplasma, using the MycoAlert detection kit (Lonza, #LT07-218).

Commonly misidentified lines
(See ICLAC register)

All cell lines used were checked against the ICLAC database of misidentified cell lines.
- COS-7 is listed as misidentified. It was used here solely for technical validation of the imaging method. Its possible misidentification does not impact the methodological goals of this study. No further authentication was performed beyond sourcing from ECACC.

- Jurkat (clone E6-1) and HepG2 (ATCC HB-8065) are not listed as misidentified. Both were obtained from ATCC, which performs STR profiling for authentication.
Authentication details are noted hereabove. No further in-house validation was performed.

Plants

Seed stocks	n.a.
Novel plant genotypes	n.a.
Authentication	n.a.
